# Tris-(2,3-Dibromopropyl) Isocyanurate, a New Emerging Pollutant, Impairs Cognition and Provokes Depression-Like Behaviors in Adult Rats

**DOI:** 10.1371/journal.pone.0140281

**Published:** 2015-10-12

**Authors:** Liang Ye, Zhengping Hu, Hui Wang, Haibo Zhu, Zhaoju Dong, Wanglin Jiang, Huijuan Zhao, Ning Li, Wei Mi, Wenyan Wang, Xihou Hu

**Affiliations:** 1 School of Public Health and Management, Binzhou Medical University, Yantai, Shandong, PR China; 2 Institute of Toxicology, Binzhou Medical University, Yantai, Shandong, PR China; 3 Medicine & Pharmacy Research Center, Binzhou Medical University, Yantai, Shandong, PR China; 4 School of Pharmacy, Yantai University, Yantai, Shandong, PR China; 5 School of Pharmacy, Binzhou Medical University, Yantai, Shandong, PR China; University of Regensburg, GERMANY

## Abstract

Tris-(2,3-dibromopropyl) isocyanurate (TDBP-TAZTO), an emerging brominated flame retardant, possesses the characteristics of candidate persistent organic pollutants and has displayed toxicity to fish and rodents. TDBP-TAZTO can pass through the blood brain barrier and accumulate in brain. However, the neurotoxicity of TDBP-TAZTO has not yet studied in rodents. We hypothesize that TDBP-TAZTO could induce the neurotoxicity in rat hippocampal neurons. The male adult rats were exposed to TDBP-TAZTO of 5 and 50 mg/kg by gavage, daily for 6 months. TDBP-TAZTO resulted in cognitive impairment and depression-like behaviors, which may be related with TDBP-TAZTO-induced hypothalamic-pituitary-adrenal axis hyperactivation, upregulation of inflammatory and oxidative stress markers, overexpression of pro-apoptotic proteins, downexpression of neurogenesis-related proteins in hippocampus, and hippocampal neurons damage in DG, CA1 and CA3 areas. Our findings suggested that TDBP-TAZTO induces significant hippocampal neurotoxicity, which provokes cognitive impairment and depression-like behaviors in adult rats. Therefore, this research will contribute to evaluate the neurotoxic effects of TDBP-TAZTO in human.

## Introduction

Brominated flame retardants (BFRs) have been widely used to reduce fire-related injury and property damage in plastics, textiles, synthetic fibers and other materials [1, 2]. However, some BFRs, such as tetrabromobisphenol-A and polybrominated diphenylethers, can accumulate in the human body and affect human health [3–5]. The accumulating data shown that some BFRs can induce neurotoxicity, immunotoxicity and endocrine toxicity in rodents [6–8]. The penta- and octa-BDE mixtures has been listed into the Stockholm Convention on Persistent Organic Pollutants (POPs) [9]. Currently, the demand for alternative BFRs, such as tris-(2,3-dibromopropyl) isocyanurate (TDBP-TAZTO), has steadily increased [2]. The annual production volume of TDBP-TAZTO in China in the 1990s was more than 500 metric tons [10]. Following the cessation of commercial production of some BFRs worldwide, the increasing production and use of TDBP-TAZTO may be expected in Europe, US and japan [9].

TDBP-TAZTO has recently been identified in soils, sediments, and earthworms is considered persistent [9, 11]. The studies showed that TDBP-TAZTO potentially causes the reproductive and endocrine toxic effects, and the impaired gas bladder inflation in zebrafish [10, 12], and induced significant liver and lung toxicity in mice [1]. Also, TDBP-TAZTO displays neuronal cell toxicity in primary cultured cerebellar granule neurons [13]. TDBP-TAZTO has been shown to bioaccumulate in carp brains, indicating that this substance can pass through the blood brain barrier [11]. TDBP-TAZTO of 2 mg/kg or 50 mg/kg were given to the adult rats by gavage for one month, and the ratios of brain/body weight were significantly increased [14]. These data suggest that TDBP-TAZTO has the potential to be neurotoxic, but much stronger evidence is required.

Depression and cognitive impairment have become major public health problems all over the world, both of which are often comorbid and closely related to the histopathological changes in hippocampus in both human and rodent models [15, 16]. The hippocampus is vulnerable to kinds of injuries such as the increased glucocorticoid release induced by hypothalamic-pituitary-adrenal (HPA) axis activation [17–19], the inflammation characterized with increased pro-inflammatory cytokines (IL-6, TNF-α and IL-1β) [20], and high oxidative stress [21, 22]. Furthermore, these factors can also induce neuronal cells apoptosis, inhibit neurogenesis, and reduce neuroplasticity in hippocampus [23–26]. Changes in dentate gyrus (DG), cornu ammonis 1 and 3 (CA1 and CA3) subregions morphology in hippocampus are associated with depression and cognitive impairment [27–29].

We hypothesized that long-term exposure to TDBP-TAZTO would induce the hippocampal neurotoxicity and the related behavior changes in adult rats. Male Sprague-Dawley (SD) rats were exposed to TDBP-TAZTO of 5 mg/kg or 50 mg/kg by gavage, daily for 6 months, which was based on prior studies [1, 14]. During the latest two weeks of the 6^th^ month, the depression-like behavior was assessed by forced swimming test (FCT), and learning and memory by Morris Water Maze (MWM) test. Then the potential effects of TDBP-TAZTO on HPA axis, inflammatory and oxidative stress markers, pro-apoptotic proteins, neurogenesis-related proteins in hippocampus, and hippocampal neurons damage, were also investigated.

## Materials and Methods

### Ethics Statement

This experiment was approved by the Ethics Committee of Binzhou Medical University (No. 013 in 2014 for Animal Ethics Approval). The local legislation regarding the ethics of animal experimentation and the guidelines for the care and use of laboratory animals were followed in all animal procedures. All surgery was performed under anesthesia, and all efforts were made to minimize suffering.

### Animals

Male SD rats (170–180 g) were obtained from Beijing Weitong Lihua Experimental Animal Centre and were acclimatized for at least 5 days before use in the experiments. All the animals were housed (5/cage) under constant environmental conditions (22 ± 2°C, 40–70% relative humidity) with a 12:12 h light/dark cycle (lights were on at 06:00 h and off at 18:00 h) and were free access to uniform food and sterile water. During this experiment, the animal’s appearance, mental status and mortality, were evaluated once daily; and body weight and food consumption was measured twice every week.

### Materials and Drugs

**Fig 1:**
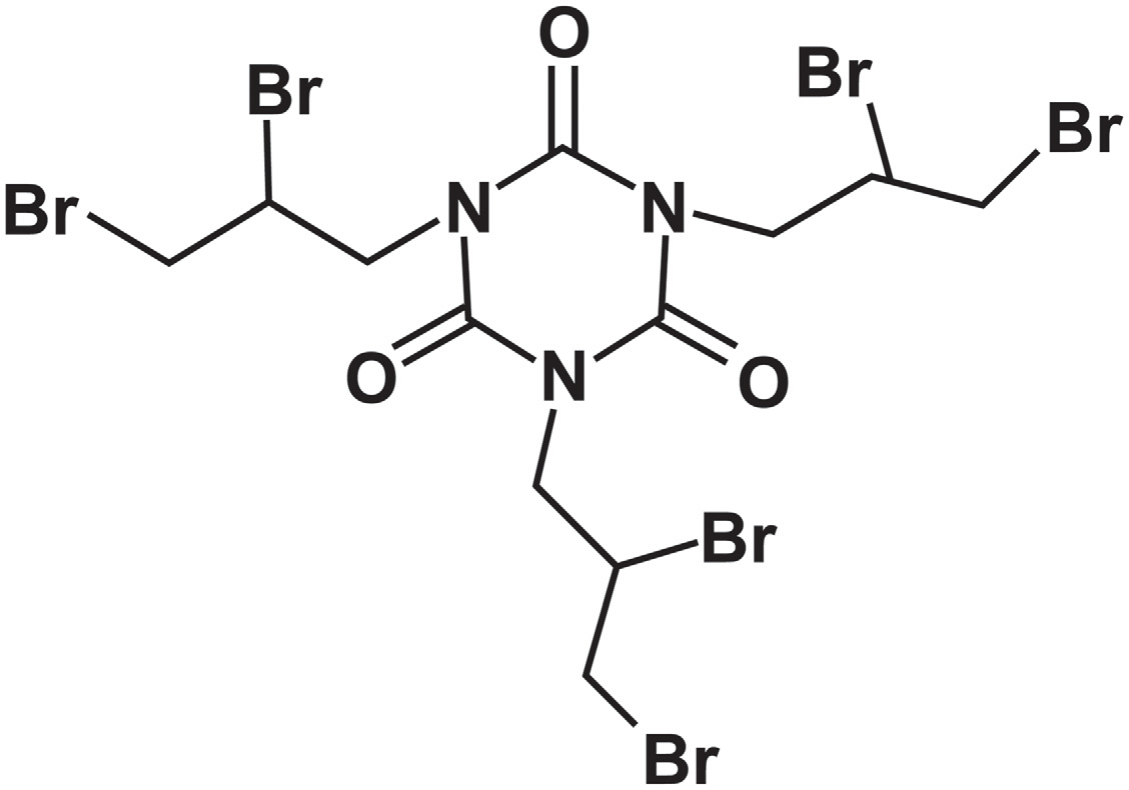
Molecular structure of TDBP-TAZTO.

The feed was provided by Shanghai SLAC Laboratory Animal Co., Ltd., (feed production certificate: Shanghai (2008)04002). TDBP-TAZTO (purity>97%, molecular structure was shown in Fig 1) was provided by Sigma-Aldrich (St. Louis, MO, USA) and dissolved in corn oil for the experiments. Rat interleukin-1 beta (IL-1β, RLB00), IL-6 (R6000B) and tumor necrosis factor-alpha (TNF-α, RTA00) ELISA kits were purchased from R&D Systems, USA. Rat brain-derived neurotrophic factor (BDNF) ELISA kit (ARG80768) was purchased from Arigo Biolaboratories Corp, Germany. The primary rabbit antibodies to Bcl-2 (Cat. no. 2876), Bax (Cat. no. 2772S) and cleaved caspase-3 (Cat. no. 9697), and mouse antibody to GFAP (Glial fibrillary acidic protein, Cat. no. 3670S), were provided by Cell Signaling Technology (Beverly, MA). Primary rabbit anti-synaptophysin (SYP, Cat. no. SAB2102352) was obtained from Sigma-Aldrich Corp. Goat anti-rabbit and goat anti-mouse IgG-HRP secondary antibodies (Cat. no. A0208 and A3216) were provided by Beyotime Biotechnology, China.

Adrenocorticotropic hormone (ACTH, Cat. no. KA0917) and corticosterone (CORT, Cat. no. KA0468) radioimmunoassay kits were obtained from Abnova Corp., USA. The assay kits of superoxide dismutase (SOD, Cat. no. S0060), maldondialdehyde (MDA, Cat. no. S0131) and glutathione (GSH, Cat. no. A006) and BCA Protein Assay kit (Cat. no. A045-3) were provided by the Nanjing Jiancheng Bioengineering Institute.

### TDBP-TAZTO Exposure

The male rats were randomly assigned into three groups (10/group). According to toxicity studies of TDBP-TAZTO in the mice and rats (1, 14), the doses were chosen as 5 mg/kg or 50 mg/kg. TDBP-TAZTO or corn oil were administrated to the rats by gavage, respectively. All the animals received a daily dose from 8 a.m. to 9 a.m. for 6 months from March 2014 to September 2014. During the latest two weeks of the 6^th^ month, the open field test, FCT and MWM test were conducted in turn.

### Open Field Test

To evaluate the possible effects of TDBP-TAZTO on locomotor activity, rats were individually placed in the plastic box (80 × 80 × 50 cm). The moving distance of rats in 20-min test was automatically recorded by a video camera and analyzed by using Topscan system (Clever Sys., Inc., USA), which was indicative of locomotor activity. The boxes were cleaned between the animals.

### Forced Swimming Test

FST was performed according to the previous method [30]. Briefly, the animal was individually forced to swim in a glass cylinder (25 cm high, 12 cm in diameter) filled with water (22 ± 2°C) to a depth of 15 cm. FST were videotaped from the side of cylinder. The total duration of immobility was measured during 6 min. Immobility was defined as floating in the water and treading water just enough to keep the nose above water [31]. The data were analyzed by using DepressionScan system (Clever Sys., Inc.).

### Morris Water Maze Test

The MWM trials were performed with a method modified from the references [32, 33]. The water maze apparatus were consisted of circular water tank (diameter 180 cm; height 60 cm) containing water at 23 ± 1°C to a depth of 30 cm. The water was made opaque by adding black ink. The pool was averagely divided into four quadrants: northeast (NE), southeast (SE), northwest (NW) and southwest (SW). The platform was submerged about 1 cm below the water surface in the target quadrant. The four trials were carried out every day for every animal, consecutively for five days (Table 1). They could freely swim to find the hidden platform. The time taken to arrive onto the platform (escape latency) was recorded. The rats which had found the hidden platform were allowed to stay on the platform for 10 s. If a rat did not find the platform within 60 s, it would be guided to reach the platform and stays for 10 s. At Day 6, a probe trial was conducted after removing the platform and the rats were allowed to swim for 60 s. The swimming time in the target quadrant were calculated. Each rat’s swimming was recorded by a video camera, and then the data were analyzed by using Topscan system.

**Table 1:** Morris Water Maze test.

	Day 1		Day 2	Day 3	Day 4	Day 5	Day 6
	Platform location	Starting Direction	Platform Location: SW; Starting Location as follows:				No platform
**Trial 1**	SW	S	W	N	N	E	N
**Trial 2**	SE	W	S	W	E	S	
**Trial 3**	NE	E	N	E	W	W	
**Trial 4**	NW	N	E	W	S	N	

Note: south (S), west (W), east (E), northwest (N), northeast (NE), southeast (SE), northwest (NW) and southwest (SW).

### Dissection and Homogenization

All the rats were anesthetized with an intraperitoneal injection of 10% chloral hydrate (300 mg/kg) between 9:00 a.m. and 11:00 a.m., and the blood samples from abdominal aorta were collected in tubes with or without heparin for serum preparation. Then, the animals were sacrificed by exsanguination from the abdominal aorta. The blood samples was collected and stored at -80°C for further tests.

The adrenal glands were rapidly removed. Brains were removed and divided at the hemisphere. The left hemispheres of ten brains per group were used for histopathological or immunohistochemical examination. And right hemispheres were placed on dry ice for hippocampus dissection and 10% (w/v) tissue homogenates were prepared in 0.1M phosphate buffer (pH 7.4) with or without protease inhibitor cocktails. The homogenates were centrifuged at 10,000g for 10 min and the supernatants were collected for the oxidative stress markers and pro-inflammatory cytokines determination, or western blotting analysis.

### Plasma ACTH and Serum CORT

Plasma ACTH and serum CORT levels were determined using the corresponding radioimmunoassay kits according to manufacturer instructions. The optical density (O.D.) was determined by using SpectraMax M5 multi-detection reader (Molecular Devices, Inc., USA).

### Oxidative Stress Markers in Hippocampus

The following oxidative stress markers in hippocampus, such as SOD activity, GSH and MDA content were determined by the biochemical assay kits according to manufacturer instructions.

### Pro-inflammatory Cytokines in Hippocampus

The total protein of hippocampus supernatants were measured using the BCA method and diluted to be the samples with the total protein of 2 mg/ml. Pro-inflammatory cytokines levels in hippocampus such as IL-1β, IL-6 and TNF-α were measured by ELISA kits according to manufacturer instructions and were expressed as pg/mg protein in hippocampus.

### Apoptosis-related Proteins in Hippocampus

The protein content of the hippocampal homogenates were measured by BCA method. The same amounts of proteins (20–30 μg per lane) were separated by SDS polyacrylamide gel electrophoresis, and transferred onto a nitrocellulose membrane. These membranes were incubated with 1% bovine serum albumin in Tween-Tris-buffered saline and reacted overnight at 4°C with the mouse or rabbits antibody (1:1000) against Bcl-2, Bax or cleaved caspase-3. After repeated washings, the immunoreactive bands were reacted with horseradish peroxidase-conjugated anti-mouse or anti-rabbit antibody, then visualized by FluorChem™ Q chemiluminescent System (ProteinSimple Corp., Canada). The O.D. velue of reactive bands visible was determined densitometrically. β-actin was used as internal control.

### BDNF and SYP Levels in Hippocampus

BDNF levels in hippocampus were determined using an ELISA kit following manufacturer instructions. SYP expression in hippocampus were assessed according to the above western blotting protocol and the primary rabbit anti-SYP antibody was diluted to 1:500.

### Histopathological Examination

The adrenal glands and five left brain hemispheres were preserved in 10% neutral phosphate-buffered formalin, then embedded in paraffin. Serial coronal sections (5-μm thickness) of brain and adrenal gland were then obtained. The paraffin sections were dewaxed and rehydrated with alcohol for HE staining. Adrenal gland and the neurons in hippocampus were examined under the inverted microscopy (CKX41, Olympus Corp., Japan).

### Immunohistochemical Examination

The five left paraffin-embedded brain hemispheres were used for immunohistochemical localization of GFAP. The sections were deparaffinized and endogenous peroxidase was blocked with H_2_O_2_ in methanol, followed by heating in 0.01 mol/L citrate buffer in a microwave for 20 minutes. After blocked with normal horse serum for 20 minute, the sections were incubated with anti-rat GFAP antibody (1:50) for 2 h. After washes, sections were overlaid with goat anti-mouse IgG-HRP secondary antibody for 30 min. The reaction was developed with 3,3-diaminobenzidine (DAB) and the sections were counterstained with hematoxylin.

### Statistical Analyses

The statistical analyses were performed using one-way ANOVA, followed by LSD post hoc test in SPSS software (V 16.0). *P* < 0.05 was considered statistically significant.

## Results

### Mortality and Clinical Signs

All the animals survived without abnormal clinical signs observed. No significant differences in food consumption and body weight gain were noted in TDBP-TAZTO and control groups (unpublished data).

### Locomotor Activity

**Fig 2:**
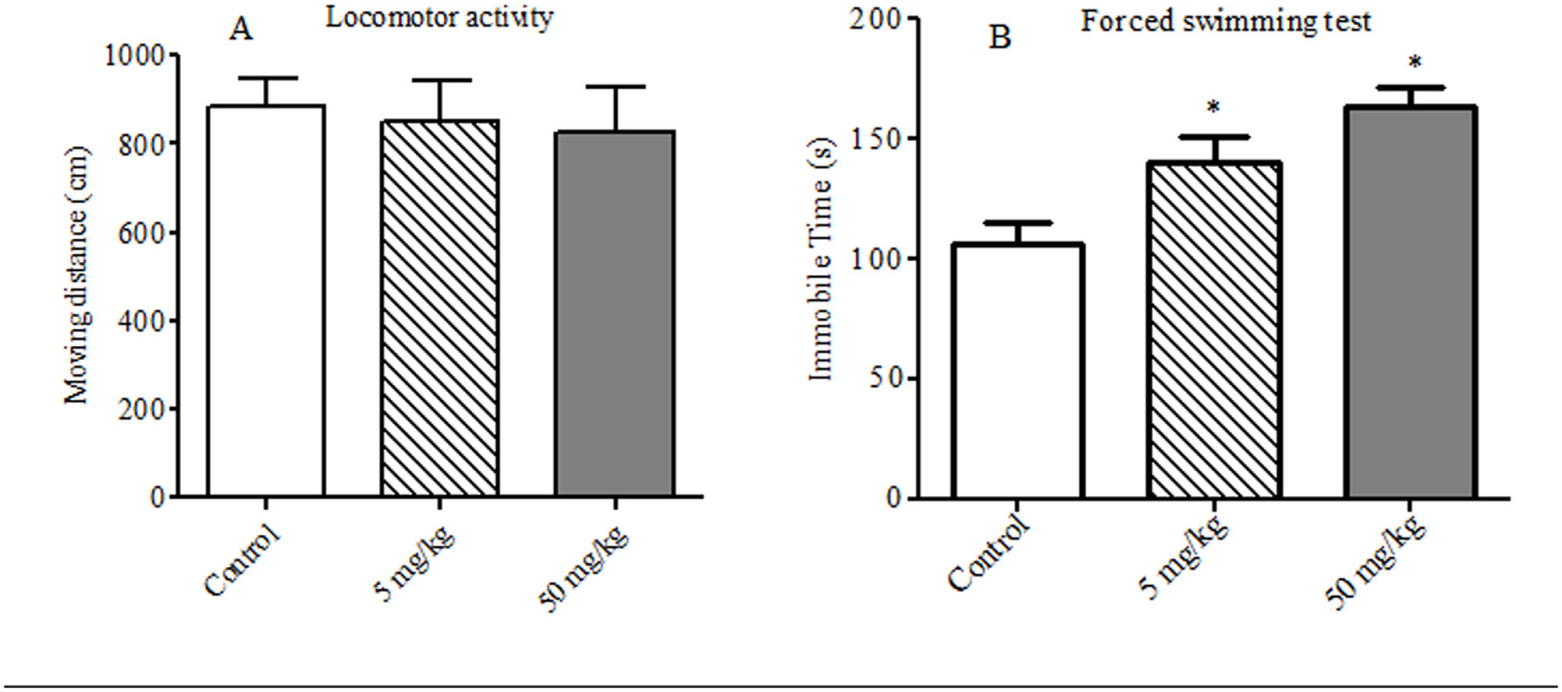
The effects of TDBP-TAZTO (5 and 50 mg/kg) on the locomotor activity (A) and the durations of immobility in FST (B) in rats. The results are presented as mean ± SE (n = 10/group). *p < 0.05, vs. control group.

Locomotor activity showed no statistical difference among the groups [F (2, 27) = 1.17, *p* = 0.325]. As seen in Fig 2A, TDBP-TAZTO of 5 and 50 mg/kg produced no significant effects on the moving distance compared with control group (each *p* > 0.05).

### Behaviors in the FST

One-way ANOVA showed statistically significant differences in the durations of immobility in FST [*F* (2, 27) = 9.83, *p* < 0.001]. Compared with control group, TDBP-TAZTO at 5 mg/kg and 50 mg/kg had significantly increased the duration of immobility in a dose-dependent manner (each *p* < 0.05) (Fig 2B).

### Learning and Memory

**Fig 3:**
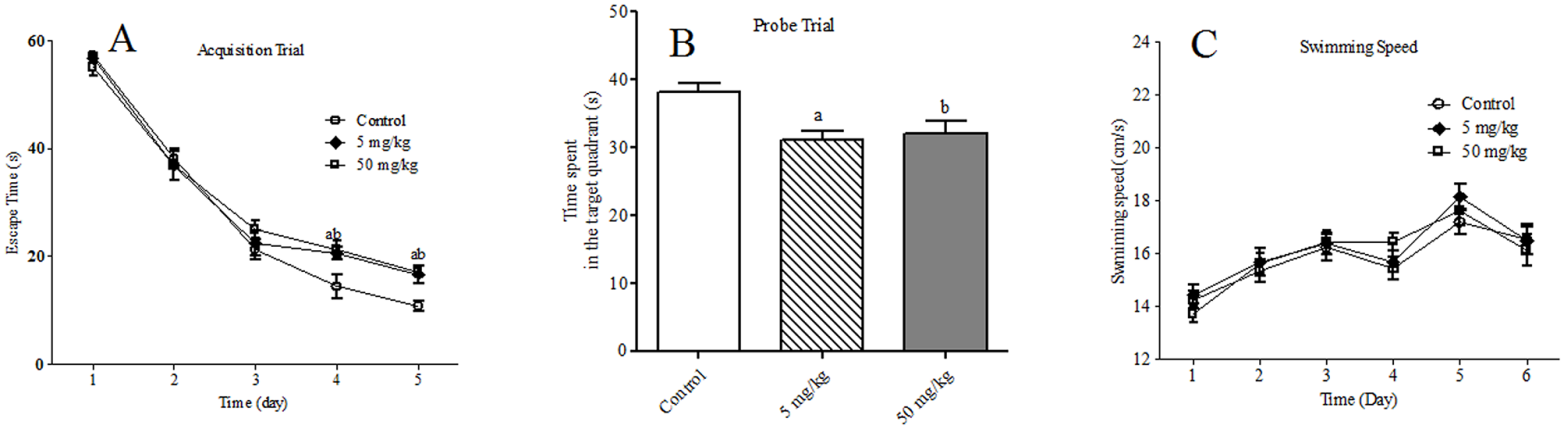
The effects of TDBP-TAZTO (5 and 50 mg/kg) on spatial learning and memory in rats in the Morris water test. (A) In the acquisition trial, TDBP-TAZTO rats showed an increased latency to the platform. (B) In the probe trial, TDBP-TAZTO rats had a decreased time spent in the target quadrant. (C) From day 1 to day 6, TDBP-TAZTO did not affect the swimming speed compared with that in control group. Data ate expressed as mean ± SE (n = 10/group). ^a^ *p* < 0.05, TDBP-TAZTO 5 mg/kg group vs. control group; ^b^ *p* < 0.05, TDBP-TAZTO 50 mg/kg group vs. control group.

In MWM test, the escape time in the acquisition trial showed statistically significant differences among the groups on the 4^th^ and 5^th^ days [*F* (2, 27) = 5.10, *p* = 0.013; *F* (2, 27) = 7.82, *p* = 0.002, respectively]. On both time points, the escape time in TDBP-TAZTO groups were significantly increased compared with that in control group (each *p* < 0.05) (Fig 3A).

In the probe trial on the 6^th^ day, the time spent in the target quadrant displayed statistically significant differences among the groups [*F* (2, 27) = 7.36, *p* = 0.003]. The time spent in TDBP-TAZTO groups were significantly lower than that in control group (each *p* < 0.05) (Fig 3B).

During the course of the experiment, on the 2^nd^, 3^rd^, 4^th^ and 5^th^ days in the acquisition trials, and the 6^th^ day in probe trial, the swimming speed were not significantly affected [*F* (2, 27) = 0.163, *p* = 0.850; *F* (2, 27) = 0.048, *p* = 0.953; *F* (2, 27) = 1.764, *p* = 0.191; *F* (2, 27) = 0.926, *p* = 0.408; F (2, 27) = 0.156, p = 0.856]. On each time points, the swimming speed in TDBP-TAZTO groups were not significantly different compared with that in control group (each *p* < 0.05) (Fig 3C). Therefore, in MWM test, TDBP-TAZTO did not affect the basic swimming ability and motivation to escape from water.

### Serum CORT and Plasma ACTH

**Fig 4:**
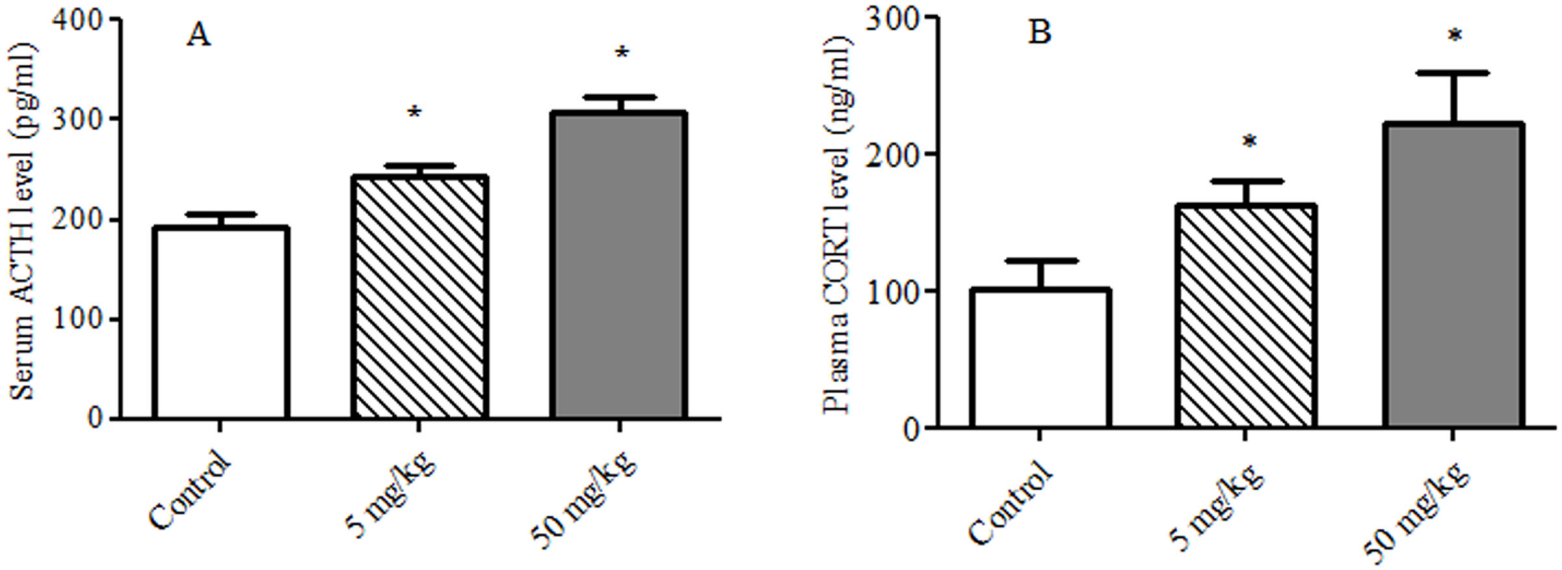
The effects of TDBP-TAZTO (5 and 50 mg/kg) on plasma ACTH (A) and serum CORT (B). The results are presented as mean ± SE (n = 10/group). *p < 0.05, vs. control group.

There were statistically significant differences in the plasma ACTH and serum CORT among the groups [*F* (2, 27) = 193.5, *p* < 0.001; *F* (2, 27) = 51.6, *p* < 0.001, respectively]. As shown in Fig 4, compared with control group, TDBP-TAZTO dose-dependently induced a significant increase in plasma ACTH and serum CORT levels (each *p* < 0.05).

### Oxidative Stress Markers in Hippocampus

MDA and GSH content and SOD activity in hippocampus showed a statistically significant differences among the groups [*F* (2, 12) = 89.99, *p* < 0.001; *F* (2, 12) = 18.34, *p* < 0.001; *F* (2, 12) = 143.3, *p* < 0.001, respectively]. As shown in Table 2, compared with control group, TDBP-TAZTO significantly increased MDA level (each *p* < 0.05), and decreased GSH level (each *p* < 0.05) and SOD activity (each *p* < 0.05) in a dose-dependent manner in hippocampus.

**Table 2:** Effects of TDBP-TAZTO on oxidative stress markers in hippocampus.

Group	MDA (nmol/mg pro)	GSH (μmol/mg pro)	SOD (U/mg pro)
**Control**	0.163 ± 0.014	0.061 ± 0.019	1.530 ± 0.101
**TDBP-TAZTO 5 mg/kg**	0.419 ± 0.092*	0.046 ± 0.009*	1.171 ± 0.109*
**TDBP-TAZTO 50 mg/kg**	0.537 ± 0.060*	0.027 ± 0.008*	0.679 ± 0.127*

Note.

*p < 0.05, vs. control group.

The results are presented as mean ± SE (n = 5/group).

### Pro-inflammatory Cytokines in Hippocampus

**Fig 5:**
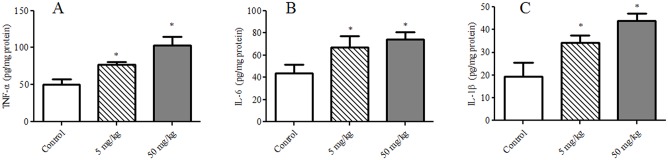
The effects of TDBP-TAZTO (5 and 50 mg/kg) on pro-inflammatory cytokines level in the hippocampus. The results are presented as mean ± SE (n = 5/group). For statistical significance, *p < 0.05, vs. control group.

One-way ANOVA revealed that there were statistically significant differences in IL-6, TNF-α and IL-1β levels in hippocampus among the groups [*F* (2, 12) = 38.41, *p* < 0.001; *F* (2, 12) = 109.10, *p* < 0.001; *F* (2, 12) = 76.75, *p* < 0.001, respectively]. TDBP-TAZTO dose-dependently increased IL-6, TNF-α and IL-1β levels compared control group (each *p* < 0.05) (Fig 5).

### Apoptosis-related Proteins in Hippocampus

**Fig 6:**
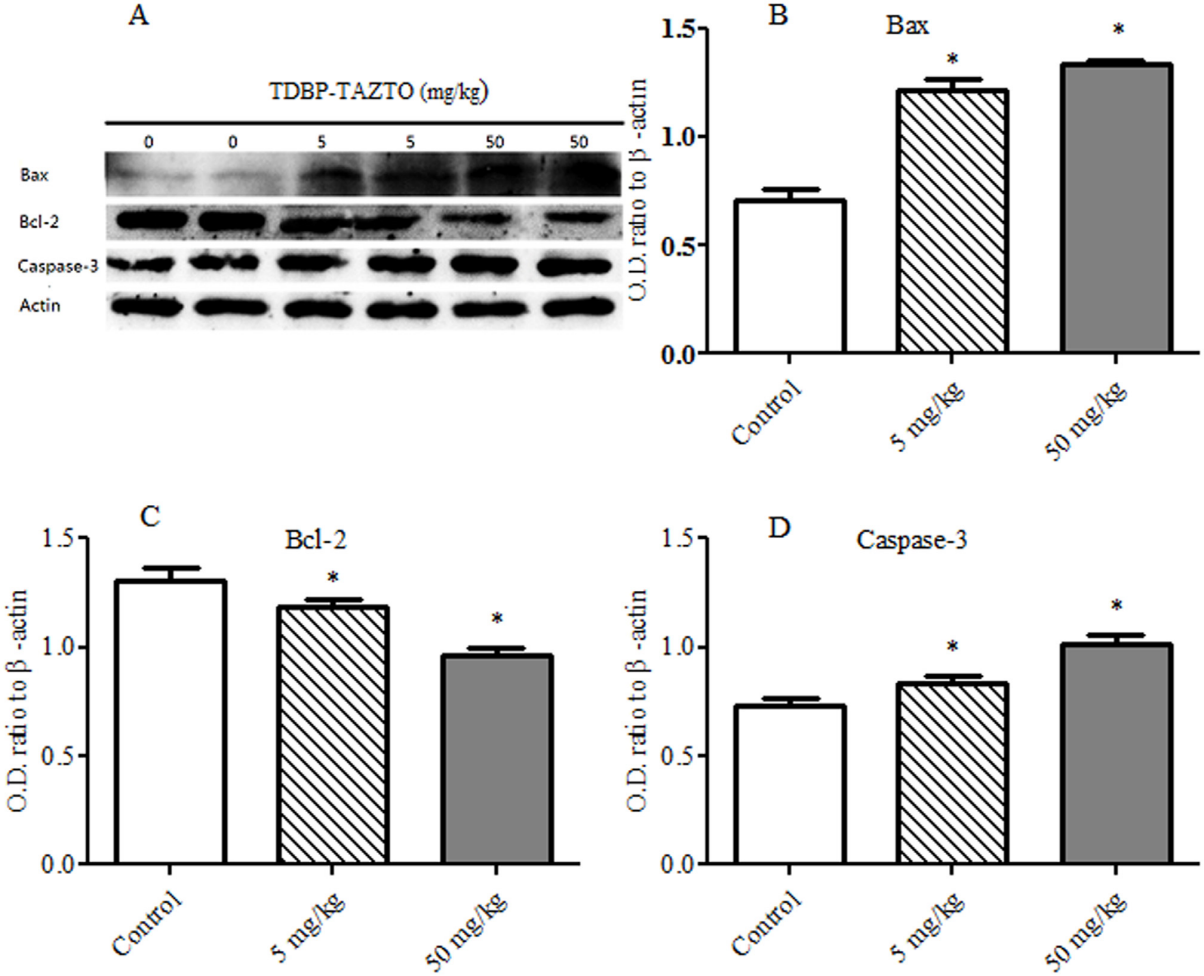
The effects of TDBP-TAZTO (5 and 50 mg/kg) on apoptosis-related proteins in the hippocampus. The results are presented as mean ± SE (n = 5/group). *p<0.05, vs. control group. The O.D. values were displayed in Bax (B), Bcl-2 (C) and cleaved caspase-3 (D) normalized to β-actin.

When studying the effects of TDBP-TAZTO on apoptosis-related proteins in hippocampus (Fig 6A), it was found that there was a main effect of TDBP-TAZTO in levels of Bax, bcl-2 and cleaved caspase-3 normalized to β-actin [*F* (2, 12) = 157.40, *p* < 0.001; *F* (2, 12) = 45.14, *p* < 0.001; *F* (2, 12) = 58.30, *p* < 0.001, respectively] (Fig 6B–6D). TDBP-TAZTO dose-dependently increased Bax and cleaved caspase-3 levels, while decreased bcl-2 levels compared to control group (each *p* < 0.05).

### BDNF and SYP Expression in Hippocampus

**Fig 7:**
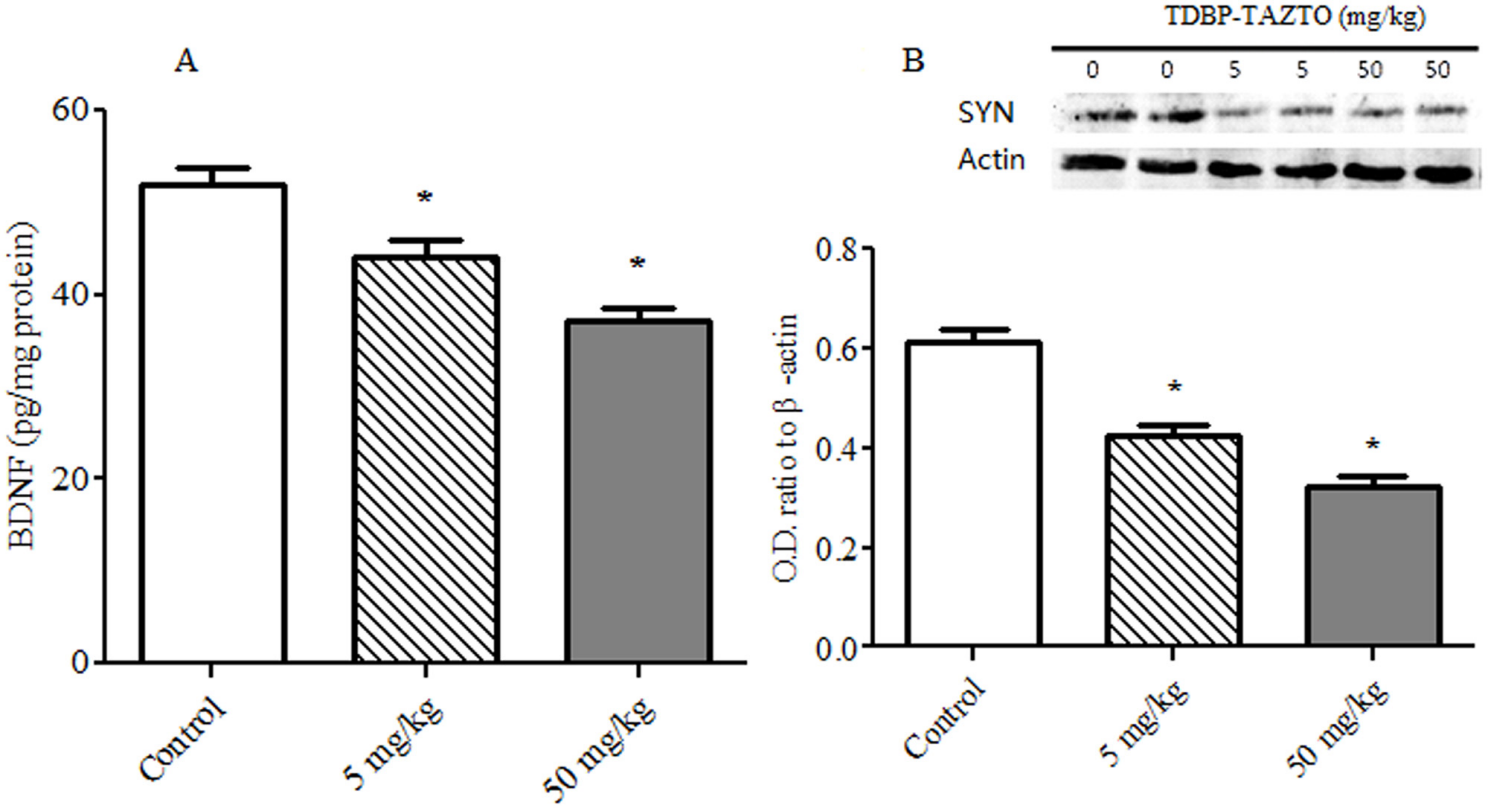
The effects of TDBP-TAZTO (5 and 50 mg/kg) on BDNF and SYP expression in hippocampus. The results are presented as mean ± SE (n = 5/group). *p<0.05, vs. control group. BDNF (A) was expressed as pg/mg protein. The O.D. values were displayed in SYP (B) normalized to β-actin.

As shown in Fig 7, TDBP-TAZTO had a significant effects on BDNF and SYP levels in hippocampus [*F* (2, 12) = 20.41, *p* < 0.001; *F* (2, 12) = 40.06, *p* < 0.001, respectively] (Fig 7A and 7B). TDBP-TAZTO dose-dependently decreased BDNF and SYP expression compared to control group (each *p* < 0.05).

### Histopathological Examination

**Fig 8:**
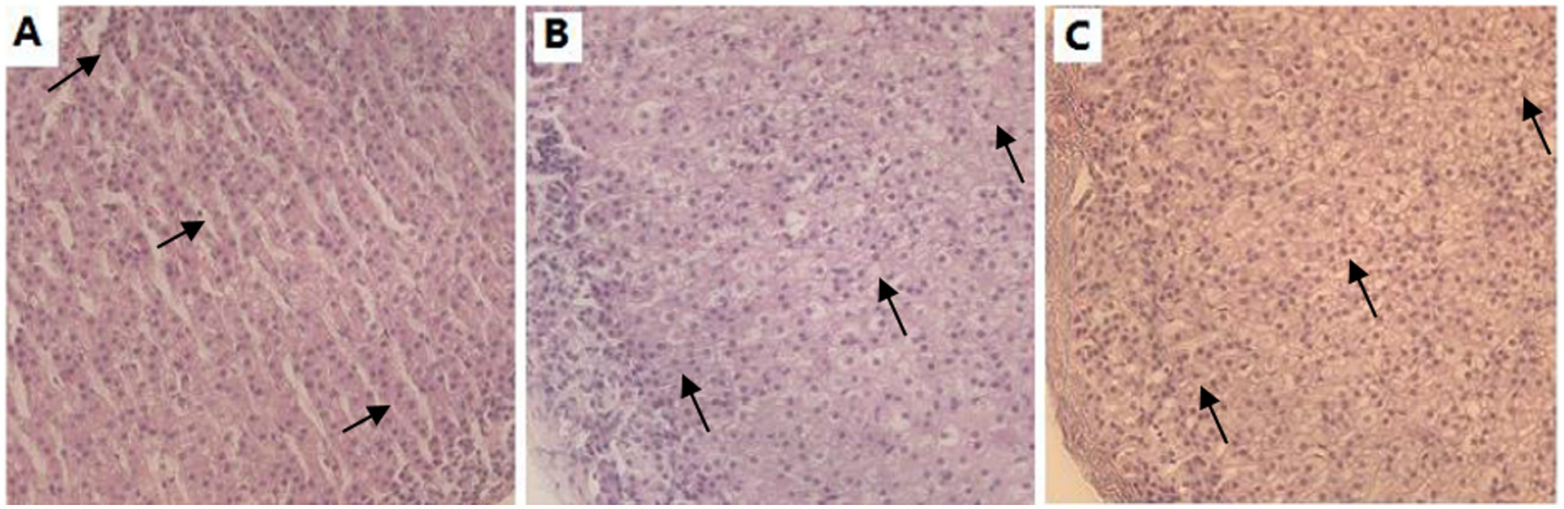
Representative HE staining of adrenal gland (200 ×). Sections from control group (A), TDBP-TAZTO 5 mg/kg group (B) and TDBP-TAZTO 50 mg/kg group (C). The arrows indicates zona fasciculata. The regularly arranged long columnar cells without cell hyperplasia or hypertrophy were observed in the zona fasciculata in the control group (A). Main diffuse adrenal hyperplasia and hypertrophy of the zona fasciculate were noted in 5 and 50 mg/kg groups (B and C).

The zona fasciculata of adrenal were occupied by regularly arranged long columnar cells without cell hyperplasia or hypertrophy in the control group (Fig 8A). However, there were main diffuse adrenal hyperplasia and hypertrophy of the zona fasciculata, which resulted in the marked increases in cortical thickness in TDBP-TAZTO 5 and 50 mg/kg groups (Fig 8B and 8C).

**Fig 9:**
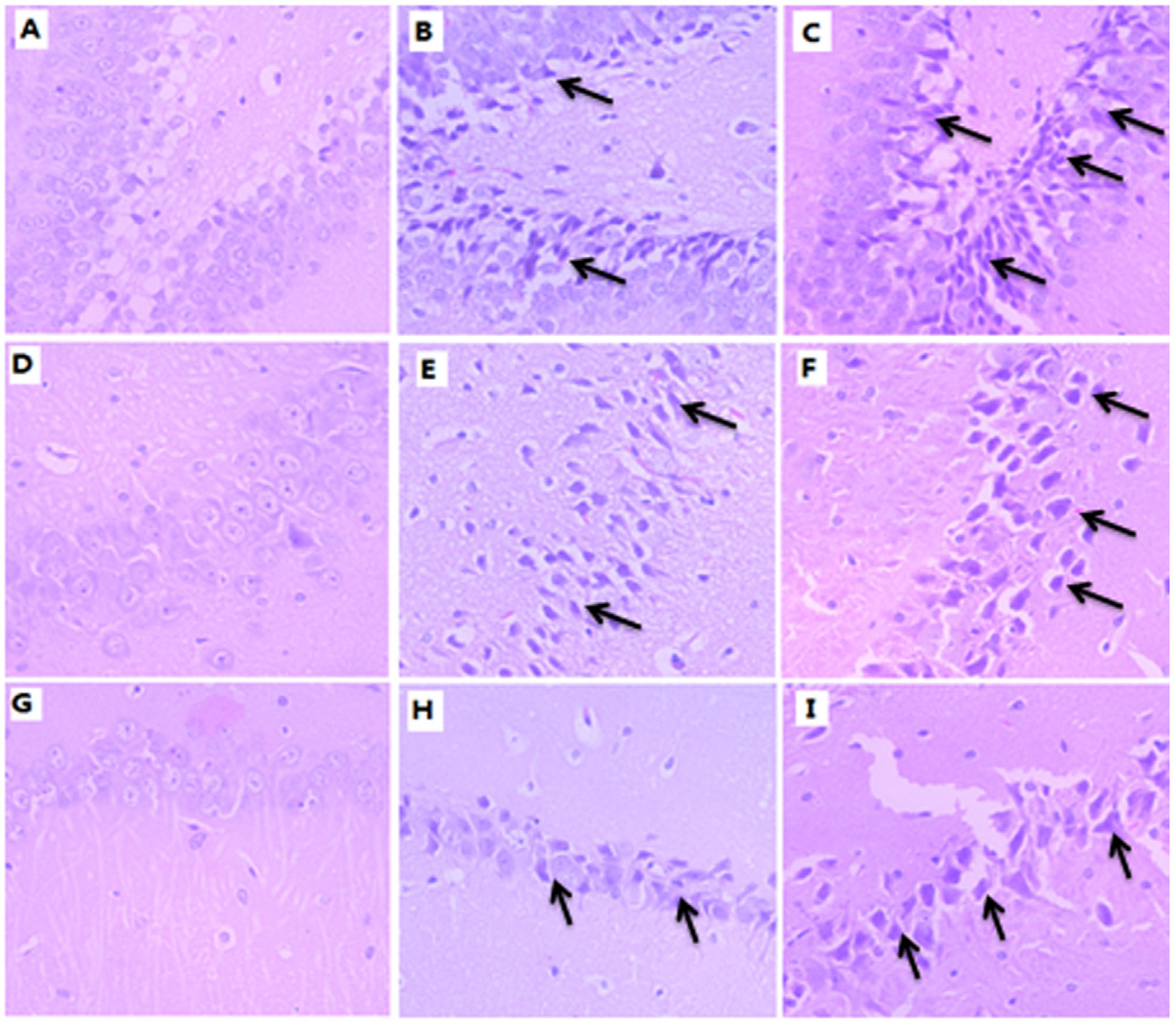
Representative HE staining of DG (A-C), CA3 (D-F) and CA1 regions (G-I) of the hippocampus (200 ×). The sections from control group (A, D and G), TDBP-TAZTO 5 mg/kg group (B, E and H) and 50 mg/kg group (C, F and I). The arrows indicates neuronal nucleus shrinkage in the DG granular neurons, CA3 and CA1 pyramidal neurons. In the control group, DG granular neurons (A), CA3 and CA1 pyramidal neurons (D and G, respectively) were observed with clear round outline, well preserved cytoplasm and distinct integrated nucleus. 5 mg/kg induced mild disorganization of DG granular cell layer (B), CA3 and CA1 pyramidal cell layers (E and H, respectively) with slight neuronal damage and nucleus shrinkage, while 50 mg/kg induced moderate-severe neuronal damage and nucleus shrinkage in these areas (CA3, CA1 and DG areas in C, F and I, respectively).

HE sections of hippocampus were observed by light microscope. In the control group, DG granular neurons (Fig 9A), CA3 and CA1 pyramidal neurons (Fig 9D and 9G) were observed with clear round outline, well preserved cytoplasm and distinct integrated nucleus. However, TDBP-TAZTO of 5 mg/kg and 50 mg/kg disrupted hippocampal cytoarchitecture. 5 mg/kg induced mild disorganization of DG granular cell layer (Fig 9B), CA3 and CA1 pyramidal cell layers (Fig 9E and 9H) with slight neuronal damage and nucleus shrinkage, while 50 mg/kg induced moderate-severe neuronal damage and nucleus shrinkage in these areas (CA3, CA1 and DG areas in Fig 9C, 9F and 9I, respectively). These results indicated that TDBP-TAZTO caused a significant increase in the number of apoptotic cells and leads to marked neuronal damage in hippocampal area.

**Fig 10:**
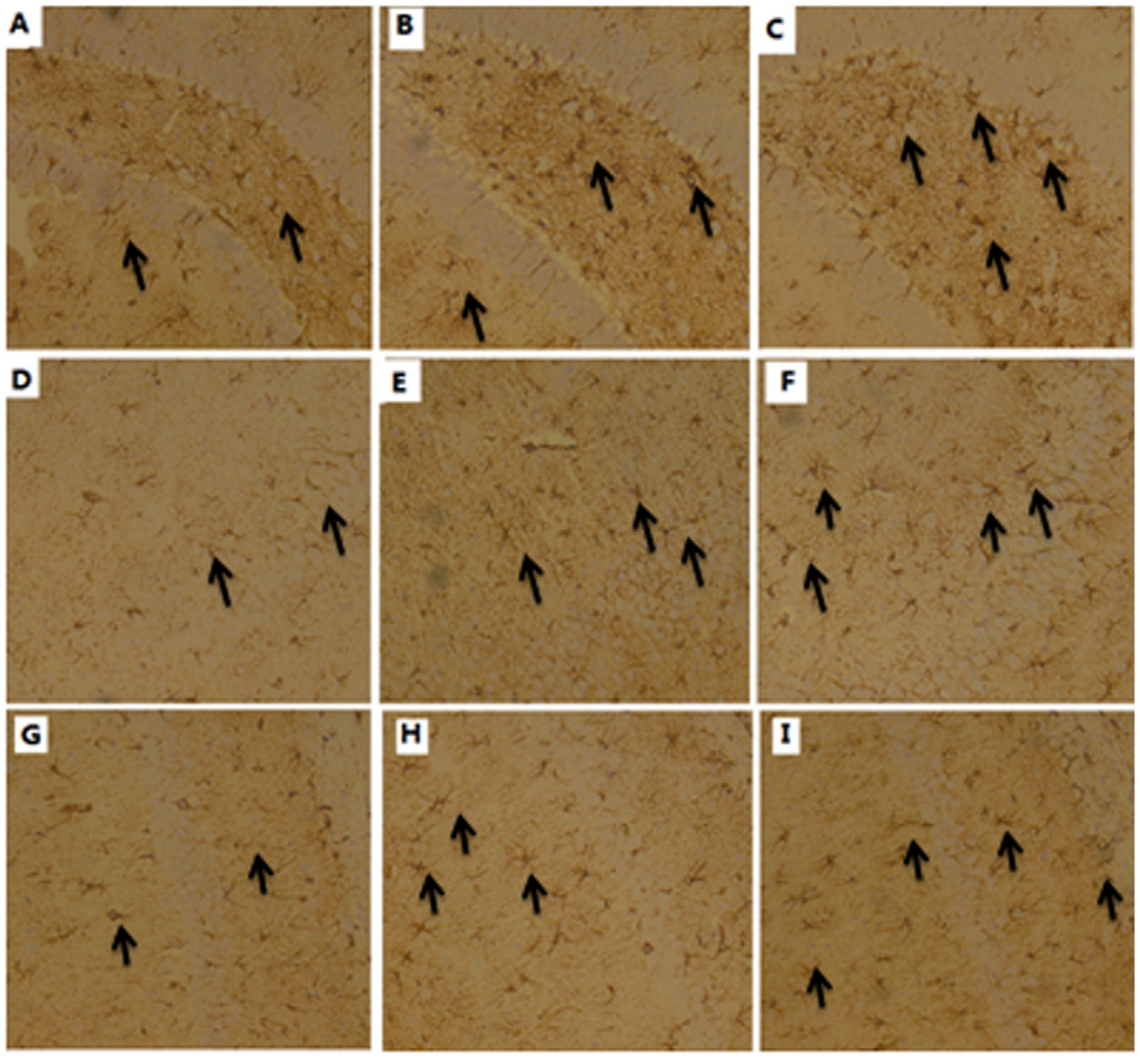
Representative immunohistochemical location of GFAP in DG (A-C), CA3 (D-F) and CA1 regions (G-I) of the hippocampus (200×). The sections from control group (A, D and G), TDBP-TAZTO 5 mg/kg group (B, E and H) and 50 mg/kg group (C, F and I). The arrows indicates astrocytes. DG, CA3 and CA1 areas had some small dispersed non-branched astrocytes in the control rats (A, D and G, respectively), while small or large branched astrocytes in 5 mg/kg (B, E and H, respectively) and 50 mg/kg rats (C, F and I, respectively).

Immunohistochemical results revealed GFAP expression of astrocytes in hippocampus in all the groups. DG, CA3 and CA1 areas had some small dispersed non-branched astrocytes in the control rats (Fig 10A, 10D and 10G, respectively), while small or large branched astrocytes in 5 mg/kg (Fig 10B, 10E and 10H, respectively) and 50 mg/kg rats (Fig 10C, 10F and 10I, respectively). Compared to the control rats, there was a significant increase in the number of astrocytes in 5 and 50 mg/kg groups.

## Discussion

BFRs have been widely applied to prevent or minimize fire hazards. The recent studies indicated that some BFRs can induce neurotoxicity, including some POPs [7]. The adverse effects of BFRs on MWM learning abilities were widely studied during early neurodevelopment [7]. However, there are no reports focusing on the neurotoxicity of BFRs to the adults. Our paper is one of initial studies to investigate the neurotoxicity of BFRs in the adult mammalian species. And, in the present study, we obtained the novel findings that TDBP-TAZTO causes depression-like behaviors and impairs cognition in the adult rats.

Because TDBP-TAZTO has recently been considered as candidate persistent organic pollutants and can bioaccumulate in some organisms such as the brain [11, 14], and may potentially harm adult health after long-term exposure, the adult rats were selected for the evaluation of its provoking depression-like behaviors and impairing cognition after exposed to TDBP-TAZTO for 6 months.

FST is widely used for evaluating depression-like activity and the immobility reflects behavioral despair similar to human depression [34]. After the exposure for 6 months, TDBP-TAZTO of 5 and 50 mg/kg significantly increased immobility time with a dose-dependent manner, but did not affect the total activity in open field test, indicating that TDBP-TAZTO did not induce motor deficits. These results demonstrated that TDBP-TAZTO induced depression-like behaviors in FST.

In MWM test, the hidden platform acquisition/learning and probe/memory trials without the platform are often used to assess spatial learning, and spatial memory after the last learning trial, respectively [35, 36]. In our study, the probe trial was carried out at 24h after the last platform learning trial, which has been a most common used method to evaluate the effects on spatial memory [35, 37]. During the acquisition trials, TDBP-TAZTO of 5 and 50 mg/kg dose-dependently impaired learning performance. Furthermore, the significantly decreased time spent in the target quadrant in the probe trial was observed in both TDBP-TAZTO groups. However, because the impaired spatial learning could confound the effects of TDBP-TAZTO on spatial memory, the changes in spatial memory could not completely be explained as the effects of TDBP-TAZTO. In order to exclude the effects of swimming speed on the acquisition/learning or memory in MWM test, the average swimming speed during the experiment were analyzed. The results found that the swimming speed of the rats did not show significant differences on the 2^nd^, 3^rd^, 4^th^, 5^th^ or 6^th^ during the acquisition phase or probe trial, suggesting that the basic swimming ability and motivation to escape from water were not affects by TDBP-TAZTO. These data suggested that long-term exposure to TDBP-TAZTO can result in cognitive impairment in adult rats.

It is well known that continuous activation of the HPA axis can induce abnormal increase of stress hormones such as CORT and ACTH, which has been considered to be closely related with cognitive dysfunction and depressive disorders in rodents and humans [17–19]. As the final secretory product of HPA activation, CORT can easily cross the blood—brain barrier and enter the brain, and influence brain functions and behavior by binding to glucocorticoid receptors in hippocampus, etc. In the present study, plasma ACTH and serum CORT levels in adult rats were significantly elevated in a dose-dependent manner following 6-month exposure of TDBP-TAZTO, indicating hyperactivation of the HPA axis. The accompanying adrenal hyperplasia and hypertrophy of the zona fasciculata in TDBP-TAZTO-treated rats also supported this result. These data suggested that TDBP-TAZTO induced cognition impairment and depression-like responses, at least in part, by upregulating ACTH and CORT levels. The published data also reported that the environmental organic contaminants including POPs can impair the stress system and activate the HPA axis [38].

The accumulating evidences show that chronic exposure to CORT can enhance neuroinflammatory and neurotoxic responses via activation of astrocytes and increase pro-inflammatory cytokine expression in hippocampus, suggesting that hyperacitvated HPA contribute to neuroinflammation in hippocampus [39–41]. Neuroinflammation and oxidative stress have been identified as the common and basic mechanisms causing CNS diseases [20, 22]. Diverse environmental factors such as POPs and air pollution can induce neuroinflammation and oxidative stress leading to CNS pathology.

The dysregulation of the control and release of pro- and anti-inflammatory cytokines is the key to neuroinflammation effects on neurodegenerative diseases and depression [20]. In our present study, we found the elevated IL-6, TNF-α and IL-1β levels in hippocampus following exposure to TDBP-TAZTO of 5 and 50 mg/kg. In addition, GFAP immunohistochemical results shown that TDBP-TAZTO increased the number of astrocytes in DG, CA3 and CA1 areas. These results suggested that TDBP-TAZTO could increase pro-inflammatory cytokines form astrocytes accompanied with neuronal damage in hippocampus.

High oxidative stress or low antioxidant status has been implicated in the pathogenesis of cognitive impairment and depression [21, 22]. The oxidative stress can cause damages to DNA and membrane fatty acids and adversely affect gene expression and proteolysis, which also contribute to CNS diseases. In this study, TDBP-TAZTO induced the oxidative stress, characterized by an increase in lipid peroxidation products and reduction in GSH content and SOD activity in hippocampal region.

Pro-inflammatory factors and oxidative stress can damage neuronal cells by increasing pro-apoptotic marker and caspase 3 activities [23]. Apoptosis has been implicated in various neurodegenerative and depression-like disorders. The anti-apoptotic protein Bcl-2 and pro-apoptotic protein Bax in the mitochondria have important effects on apoptosis. The ratio of Bcl-2/Bax is a crucial factor in determining the progress of cell apoptosis. After apoptotic signaling events have occurred, the activation of Caspase-3, a key mediator of the apoptotic cell death, is necessary in the apoptosis pathways, which would kill neuronal cells. In this study, the decreased ratio of Bcl-2/Bax and the increased caspase-3 activity in rat hippocampus were induced by TDBP-TAZTO of 5 and 50 mg/kg. Furthermore, the histopathological results shown that TDBP-TAZTO increased the number of apoptotic neuronal cells in DG, CA3 and CA1 areas. These results suggested that TDBP-TAZTO could induce hippocampal neuronal cells apoptosis directly or by activating astrocytes.

In hippocampus, BNDF can support neuronal survival, differentiation, function and plasticity, and SYP is an indicator of synaptic density. The exposure to kinds of stress, such as the hyperactivated HPA axis and excessive pro-inflammatory cytokines, can decrease BDNF and SYP expression, which reduces neuroplasticity and induces atrophy in the hippocampus in the patients with CNS diseases [24–26]. Our results showed that TDBP-TAZTO of 5 and 50 mg/kg markedly reduces BDNF and SYP expression in hippocampus, suggested that TDBP-TAZTO can impair neurogenesis and neuroplasticity, which was related with the hyperactivated HPA axis and excessive pro-inflammatory cytokines induced by TDBP-TAZTO.

One drawback of this study is that the effects of TDBP-TAZTO were only evaluated in adult male rats. Future studies investigating the effects of TDBP-TAZTO exposure on the female population are warranted. Furthermore, the effects of developmental TDBP-TAZTO exposure are also being planned.

In summary, this study demonstrated that 6-month TDBP-TAZTO exposure induced the neurotoxicity in adult rat hippocampal neurons for the first time. We found that TDBP-TAZTO may result in HPA axis hyperactivation, upregulation of inflammatory and oxidative stress makers, overexpression of pro-apoptotic proteins, downexpression of neurogenesis-related proteins in hippocampus, and hippocampal neurons damage in DG, CA1 and CA3 areas, which may have contributory roles in cognitive impairment and depression-like behaviors. Our results provide reference data regarding TDBP-TAZTO-induced neurotoxicity in adult rodents and contribute to evaluating the application of TDBP-TAZTO in the ambient environment.

## Supporting Information

S1 Table The Guidelines Checklist for Animal Research: Reporting In Vivo Experiments (ARRIVE). The SD rats were used for the in vivo experiments, all the related information was provided.
(PDF)
